# Agrarian Diet Improves Metabolic Health in HIV-positive Men with *Prevotella*-Rich Microbiomes: Results from a Randomized Trial

**DOI:** 10.21203/rs.3.rs-5349309/v1

**Published:** 2024-11-15

**Authors:** John B. O’Connor, Jennifer Fouquier, Charles P. Neff, John D. Sterrett, Tyson Marden, Suzanne Fiorillo, Janet C. Siebert, Jennifer Scheinder, Nichole Nusbacher, Amy T. Noe, Blair Fennimore, Janine Higgins, Thomas B. Campbell, Brent Palmer, Catherine Lozupone

**Affiliations:** 1. Department of Microbiology & Immunology, University of Colorado Anschutz Medical Campus, Aurora, CO, USA; 2. Department of Biomedical Informatics, School of Medicine, University of Colorado Anschutz Medical Campus, Aurora, CO, USA; 3. Computational Bioscience Program, University of Colorado Anschutz Medical Campus, Aurora, CO USA; 4. Department of Medicine, University of Colorado Anschutz Medical Campus, Aurora, CO, USA; 5. Department of Integrated Physiology, University of Colorado Boulder, Boulder, CO USA; 6. CytoAnalytics, Denver, CO, USA

## Abstract

This study aimed to assess the impact a high-fiber/low-fat agrarian diet (AD) on inflammation and metabolic outcomes in HIV positive men who have sex with men (MSM). Since the gut microbiome of MSM has been shown to have a striking resemblance to individuals in agrarian cultures, including being *Prevotella*-rich and *Bacteroides*-poor, we hypothesized that they would have particularly strong health benefits from consumption of a diet matched to their microbiome type. Sixty-six participants, including 36 HIV-positive MSM (HIV(+)MSM), 21 HIV-negative MSM, and 9 HIV negative men who have sex with women were randomized to either an AD or a high-fat western diet (WD) for four weeks. The AD reduced low-density lipoprotein cholesterol in HIV(+)MSM, with more significant reductions predicted by *Prevotella*-rich/*Bacteroides*-poor microbiomes at baseline. The AD also reduced T cell exhaustion and pro-inflammatory intermediate monocytes and altered host transcription in the colonic mucosa. Our findings suggest that tailoring diet interventions to baseline microbiome type can help promote metabolic health in HIV(+)MSM.

Despite effective preventative measures, thousands of new human immunodeficiency virus type 1 (HIV-1) infections occur each day globally.^1–3^ Although anti-retroviral therapy (ART) has improved the lifespan of people living with HIV (PLWH), these individuals still have elevated mortality and higher incidence of metabolic and cardiovascular disease (CVD).^4–10^ Translocation of gut bacteria and bacterial products across intestinal epithelial mucosal barriers contributes to chronic immune activation and systemic inflammation, which are linked to metabolic co-morbidity in PLWH. ^11–17^ Characterizing the biological mechanisms underlying inflammatory and metabolic diseases in PLWH has promise for developing treatment strategies.

HIV status is associated with gut microbiome characteristics, including lower diversity and decreased commensal bacteria.^18–22^ In the US and Europe, HIV-positive and HIV-negative men who have sex with men (MSM) have gut microbiomes characterized by higher *Prevotella* and lower *Bacteroides* relative abundance compared to men who have sex with women (MSW) and women.^23,24^ Comorbidities associated with HIV, including metabolic dysfunction and CVD, correlate with gut microbiome dysbiosis and inflammation, underscoring a complex interplay between the gut microbiome, inflammation, and HIV-associated metabolic dysfunction.^6–8,25–29^

Diet, a driver of gut microbiome variation, can potentially reduce negative clinical manifestations.^30^ A *Prevotella*-rich/*Bacteroides*-poor (PRBP) microbiome is characteristic of healthy HIV-negative individuals from African and Amerindian cultures ingesting agrarian diets (AD) high in fiber and HIV-negative participants in the US ingesting high-fiber/low-fat diets.^22,31–33^ However, HIV-positive and HIV-negative MSM exhibit PRBP microbiomes even when ingesting high-fat/low-fiber diets. ^24^ The PRBP microbiome of MSM resembles those in agrarian cultures, having high alpha diversity and high strain-level variation in *Prevotella* species.^22,34^ Reportedly, high-fiber diets have beneficial metabolic responses in humans with *Prevotella*-rich microbiomes, and high-fat diets confer pro-inflammatory responses and accelerated disease progression in macaques infected with simian immunodeficiency virus (SIV).^35,36^ These findings suggest that, with a high-fat western diet (WD), the prevalence of a PRBP microbiomes in MSM with positive HIV status may result in a diet/microbiome mismatch, contributing to inflammatory and metabolic disease. An AD that matches the microbiome can potentially mitigate HIV-associated co-morbidities.

This study assessed the effect of short-term diet modification on inflammatory/metabolic-disease markers in HIV-positive MSM on ART and HIV-negative MSM and MSW, with the hypothesis that AD would reduce inflammatory/metabolic disease markers in HIV-positive MSM with PRBP microbiomes. Participants consumed either an AD or a control WD, which mimicked a typical US diet. Investigating the effects of AD on microbial composition, blood inflammatory markers, blood and intestinal immune cell populations, and metabolic health markers provides insight into the use of dietary interventions in mitigating negative clinical manifestations in PLWH.

## Results:

### Study Cohorts Were Comparable at Baseline:

Sixty-six individuals were enrolled, including 36 (54.5%) HIV-positive MSM (HIV(+)MSM), 21 (31.8%) HIV-negative MSM (HIV(−)MSM), and 9 (13.6%) HIV-negative MSW (HIV(−)MSW) (Table 1). HIV(+)MSM participants were older than the HIV(−)MSW (p=0.010). All HIV(+)MSM were on effective ART, which in most participants was two nucleoside reverse transcriptase inhibitors (NRTIs) in combination with an integrase strand transfer inhibitor (INSTI; 67.6%) or non-nucleoside reverse transcriptase inhibitors (NNRTIs; 17.6%).

### AD Resulted in Expected Dietary Changes:

Participants, after randomization to WD or AD, completed a 4-week diet intervention, including two weeks of prepared meals and two weeks of guided self-feeding. The WD arm had a target content of fat, fiber, sugar, sodium, and carbohydrates approximating typical diets in the United States and other Western countries (Table 2). The AD was designed to mimic the diet of agrarian African cultures which are much higher in total fiber and lower in fat, protein, sugar and sodium.^37^

**Figure 1:**
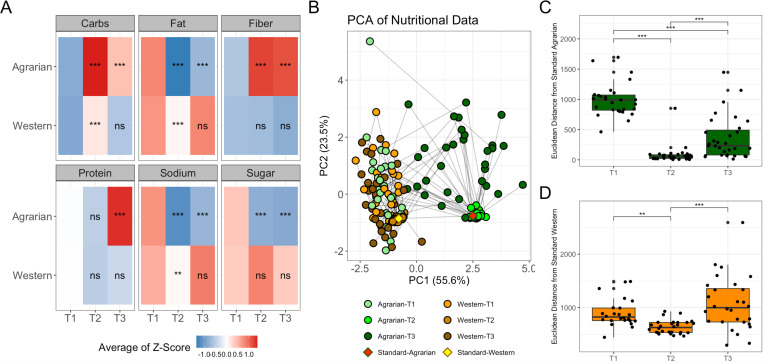
AD intervention Associated with Dietary Change Towards Target Macronutrient Intake. (A) Heatmap of dietary components at baseline (T1), 2-weeks (T2) and 4-week (T3) for individuals on an agrarian or western diet intervention. Heatmap is colored using the average log-midpoint transformed values for carbohydrates, fat, fiber, protein, sodium, and sugar normalized to 1000 kcals. Significance for dietary composition levels between timepoints was tested using a paired nonparametric Friedman test with a Bonferroni correction for multiple comparisons, to compare both T2 and T3 to T1/baseline and is indicated by the following symbols: (B) Principal Component Analysis (PCA) plot of Euclidean distances between macronutrient totals estimated from reported nutritional data including protein, fat, carbohydrates, fiber, sodium, and sugar. Points are colored by diet intervention arm, and timepoint (Baseline=T1, two weeks=T2, and four weeks=T3). The target macronutrient compositions (averages of T2) are also plotted as “Standard-Agrarian” and “Standard-Western”; Distances from the standard target diet for those on (C) AD and (D) WD with brackets indicating statistics determined by Wilcoxon-signed rank test *-p< 0.05, **-p< 0.01), ***-p< 0.001. PCA=Principal components analysis, PC=principal component, ns=not significant.

Diet was assessed at baseline (T1) using the questionnaire as described in the methods.^38^ Diet compliance after the first two weeks (T2) of prepared food was monitored during meal-pickup when uneaten food and any extra food consumed were recorded. Compliance after the second 2-week period of self-prepared diet (T3) from a set menu was monitored by 24-hour food recall. Total carbohydrates, fat, fiber, protein, sodium, and sugar were estimated at all timepoints. AD was associated with elevated carbohydrates and fiber and decreased fat, sodium, and sugar at T2 compared to baseline, and to a lesser extent at T3 (Fig. 1A). WD-associated changes from baseline included elevated carbohydrates, reduced fat, and reduced sodium at T2, and no significant changes from baseline at T3. Those on the WD still had higher fat (p=2.98×10^−10^) and lower carbohydrates (p=9.07×10^−11^) than those on AD at T2.

A principal component analysis (PCA) plot based on total macronutrients per 1000 kCals shows that T1 nutritional intake was consistent with the WD target (Fig. 1B). The AD conferred more considerable dietary difference from baseline at T2 (p=1.05×10^−8^) and T3 (p=0.006) compared to the WD as measured by Euclidean distance from baseline nutritional intake. Participants showed reduction in Euclidean distance to the target diet compositions at T2 and to a lesser extent T3, with variability lower in T2, indicating increased compliance at T2 when meals were prepared, but still maintained diets during the 2 weeks of self-preparation (Fig. 1C and 1D).

### AD Reduced Lipoprotein Cholesterol:

Metabolic markers were evaluated from a fasting blood sample to assess the impact of diet intervention on metabolic health. These included triglycerides, Homeostatic Model Assessment for Insulin Resistance (HOMA-IR; calculated using fasting blood glucose and insulin levels), high-density lipoprotein cholesterol (HDL-C) and low-density lipoprotein cholesterol (LDL-C). No significant differences were observed in these measures between cohorts at baseline (Table 1), although there were trends towards lower HDL-C and higher LDL-C, triglycerides and HOMA-IR in the HIV(+)MSM, which is consistent with more extensive epidemiologic studies.^39–41^ Metabolic health measures did not differ across ART regimens.

**Figure 2:**
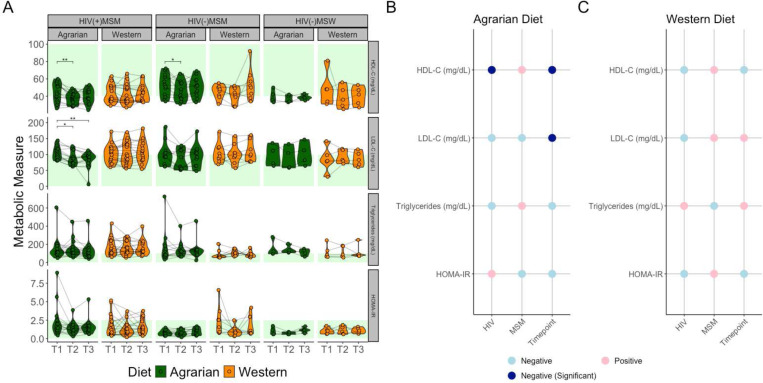
Reduced Plasma Cholesterol Levels in MSM Participants on the Agrarian Diet. (A) Violin plots overlaid with spaghetti plots of metabolic health markers colored by diet with brackets indicating significance as determined by Friedman tests with Bonferroni multiple comparisons correction; *-p≤0.05, **-p≤0.01, ***-p≤0.001, ****-p≤0.0001. Healthy ranges for each measure by previously established thresholds are indicated with green shading. ^42–44^ Panels (B) and (C) show coefficients of linear mixed-effects models relating metabolic health markers to MSM status, HIV-infection status, and timepoint in (B) those on the AD and (C) those on the WD. Red indicates a positive relationship with HIV-infection, MSM-status or change over time with the diet intervention, while blue indicates the opposite. Darker blue in Panel B represents statistical significance at p<0.05. P-values determined by analysis of variance (ANOVA) of full model (Metabolic Marker ~ HIV+MSM+(1|StudyID) vs model removing predictor of interest. HIV=human immunodeficiency virus, MSM=men who have sex with men, MSW=men who have sex with women, HDL-C=high-density lipoprotein cholesterol, LDL-C=low-density lipoprotein cholesterol, HOMA-IR=Homeostatic Model Assessment for Insulin Resistance.

Longitudinally, the AD was associated with reduced HDL-C in both MSM groups at T2 that was not sustained at T3 (Fig. 2A). In HIV(+)MSM, the AD reduced LDL-C at T2 and T3. No significant changes were observed in HOMA-IR or triglycerides. Linear mixed-effects models (LMEMs) confirmed that LDL-C and HDL-C were negatively related to time on the AD, with HDL-C also being negatively related to HIV-infection, as previously reported(Fig. 2B). ^40^ No relationships with time were observed on the WD (Fig. 2C). LMEMs revealed HDL-C (padj=9.42×10^−5^) and LDL-C (padj=4.47×10^−3^) were positively related to this distance to the standard AD, indicating more agrarian diets resulted in lower LDL-C and HDL-C.

Participants in the healthy range of these metabolic measures mostly remained in the healthy range after AD (Fig. 2A). Logistic regression revealed that individuals with healthy baseline metabolic values had the highest probability of being in the healthy range at later timepoints (Fig. S1A). This coincided with strong correlations between baseline values and the changes in those values. Lower baseline values correlated with increases and higher baseline values correlated with decreases (Fig. S1B). Because of this strong impact of baseline values, the analyses were repeated only including individuals whose metabolic measures were in the unhealthy range at baseline, finding only LDL-C reduction from T1 to T3 (padj=0.014) to be significant. Therefore, unhealthy HDL-C levels in the lower range were not exacerbated by either diet.

### AD Reduced Inflammatory Markers in Individuals with Elevated Baseline Values:

We evaluated the impact of the diet intervention on plasma inflammatory markers C-reactive protein (CRP), and interleukin-6 (IL-6), and on intestinal permeability using lipopolysaccharide binding protein (LBP) as a marker. These markers also showed no significant differences across cohorts at baseline. However, CRP and IL-6 trended higher in the HIV(+)MSM as would be expected based on prior studies (Table 1).^45^ Minimal changes in these measures were observed (Fig. S2A). IL-6 decreased from T1 to T3 in HIV(+)MSM on the AD but statistical significance was not achieved (padj=0.060). LBP was reduced from T1 to T2 in the WD in HIV(+)MSM (padj=0.023). LMEMs identified no significant relationships between inflammatory markers and time, HIV-infection status, or MSM status when stratifying by diet (Fig. S2B and C). No inflammatory measures were correlated with distance from the standard AD. Like the metabolic markers, inflammatory marker changes depended on baseline data (Fig. S2D), with higher baseline values correlating with greater reduction. In samples from participants with high baseline values (top 50th percentile), AD was associated with a reduction in IL-6 at T2 (padj=0.008) that was somewhat sustained at T3 (padj=0.058) (Fig. S2E).

### AD Reduced Immune Activation and T-Cell Exhaustion:

**Figure 3:**
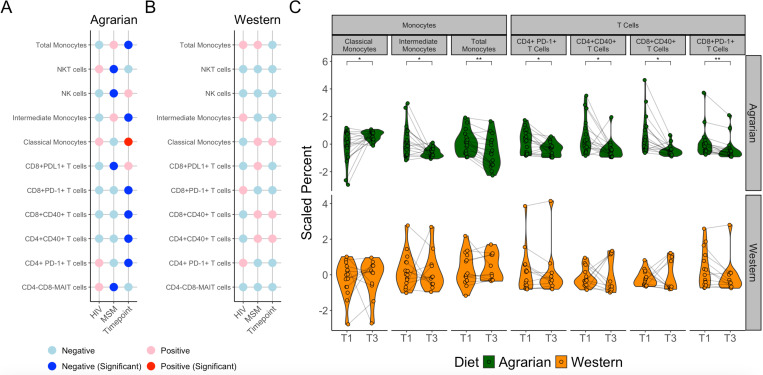
Blood Immune Cell Populations Change with Diet Intervention. (A and B) Coefficients of linear mixed-effects models (LMEMs) relating biopsy immune cells to MSM status, HIV-infection status, and timepoint in (A) those on the agrarian diet and (B) those on the western diet. Red indicates a positive relationship with positive HIV-infection status, MSM status, and change between T1 and T3 with the diet intervention, while blue indicates the opposite. Dark red and blue in panel A represent significance at p<0.05. P-values determined by analysis of variance (ANOVA) of full model (Immune Cell Population ~ HIV+MSM+(1|StudyID) vs model removing predictor of interest. Cells displayed only include those with some significant relationship on the AD. There were no cell populations significant on the western diet (WD) (C) Violin plots overlaid with spaghetti plots of blood immune cell types that correlated with time in subjects on the AD. Asterisks indicate P-values determined by ANOVA of LMEMs with FDR correction for multiple comparisons; *-p≤0.05, **-p≤0.01. NKT=natural killer T, NK=natural killer, MAIT=mucosal-associated invariant T, HIV=human immunodeficiency virus, MSM=men who have sex with men.

To characterize AD-associated immune cell differences, cytometry by time-of-flight (CyTOF) was performed on peripheral blood mononuclear cells (PBMCs) collected at T1 and T3. The monoclonal antibody (mAb) panel (Table S1) targeted diverse immune populations with a CD4+ and CD8+ T cell focus. The panel also identified populations of monocytes, macrophages, dendritic cells, B cells, Natural Killer (NK) cells, NK T cells and Mucosal-associated invariant T cells (MAIT). A representative gating strategy is shown in Fig. S3. Specific blood immune cell populations that changed on the AD were identified using LMEMs, revealing a significant decrease in CD14+ cells (total monocytes), CD14+CD16- cells (classical monocytes), CD14+CD16+ cells (intermediate monocytes), CD8+ PD-1+ T cells, CD4+ PD-1+ T cells, CD8+CD40+ T cells, and CD4+ CD40+ T cells in those randomized to the AD but not the WD arm (Fig. 3). Additionally, the AD but not the WD was associated with a significant increase in phagocytic classical monocytes and decrease in inflammatory intermediate monocytes (Fig. 3).

### Baseline Fecal Microbiome was Predictive of AD LDL-C Response:

**Figure 4:**
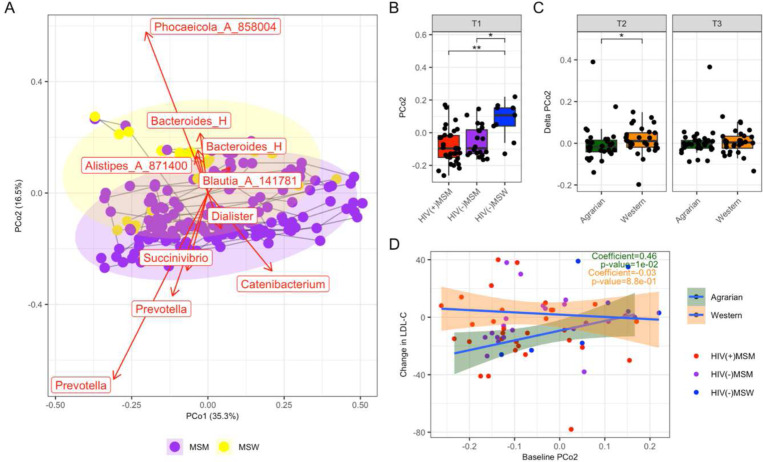
Fecal Microbiome Beta Diversity Analysis. (A) Principal Coordinates Analysis (PCoA) of Weighted UniFrac values of fecal 16S rRNA data. Points are colored by timepoint (T1=baseline, T2: after 2 weeks prepared diets, T3: after an additional 2 weeks of self-prepared meals) and Western versus Agrarian diet arm. Samples from the same individual over time are joined with a grey line. Bacterial genera are plotted in the same space using the biplots functionality of QIIME 2.^51^ The length of the arrows indicates the strength of the relationship with PCoAs 1 and 2. (B) PCoA 2 values for the 3 cohorts at baseline. (C) Changes in PCoA 2 from T1 to T2 (left panel) and T1 to T3 (right panel) Statistical significance across three groups was determined by Kruskal Wallace and pairwise comparisons made using Dunn’s post-hoc test with Bonferroni correction for multiple comparisons. (C) Linear Regression of change in LDL-C from T1 to T3 versus values of PCo2 at baseline. PCo=Principal coordinates analysis axis, MSM=men who have sex with men, MSW=men who have sex with women, HIV=human immunodeficiency virus, LDL-C=low density lipoprotein cholesterol.

To evaluate the intestinal microbiome, we performed 16S ribosomal RNA (rRNA)-targeted sequencing of fecal samples across all timepoints. Principal Coordinates Analysis (PCoA) of weighted UniFrac values showed that the fecal microbiomes separated based on relative abundances of *Prevotella* versus *Bacteroides* and *Phocaeicola* (formerly *Bacteroides*) along PCo2 (Fig. 4A).^46^ As previously reported, MSM differed from MSW by having relatively PRBP microbiomes (lower PCo2 values) (Figure 4A and B). ^23,24^ ANCOM-BC analyses of baseline bacterial genera supported these compositional differences with MSM-status, such as a significant depletion of *Bacteroides* and *Phocaeicola* (Fig. S4A).^47^ Permutational multivariate analysis of variance (PERMANOVA) testing of the weighted UniFrac distance matrix also showed a significant effect of MSM status (R^2^=0.044, p=0.001), with no significance by diet or HIV status. ANCOM-BC detected many significantly different genera in baseline samples of HIV(+)MSM compared to HIV(−)MSM (Fig. S4B) and the HIV(+)MSM had somewhat reduced Shannon diversity compared to HIV(−)MSM (p=0.069).

No AD-associated changes in the fecal microbiome were observed (Figure S4C). Samples showed robust clustering by individuals in weighted UniFrac PCoA (R^2^=0.730, p=0.001), consistent with other studies showing high interpersonal variation.^33,48–50^ Weighted UniFrac distances from baseline were not higher in the AD than the WD arm in any cohort (Fig. S4D and E). MSM did have larger changes from baseline compared to MSW in both the AD and WD arms, suggesting higher volatility of the MSM microbiomes independent of diet. We observed AD-associated reduction in PCo2, which separated individuals based on *Prevotella* and *Bacteroides* and *Phocaeicola*, from T1 to T2 compared to those on the WD, suggesting a slight shift towards a PRBP microbiome type upon initial dietary intervention, with significance lost from T1 to T3 (Fig. 4C).

To assess the effect of dietary matching, we compared baseline PCo2 and changes in LDL-C. Strikingly, individuals whose fecal microbiomes had lower values of PCo2 at baseline (i.e. more PRBP) had more significant reductions in LDL-C from T1 to T3 (Spearman p=0.010) (Fig. 4D). A more significant reduction of LDL-C with the AD was also strongly associated with elevated baseline relative abundance of *Prevotella* (Spearman p=0.009) and reduced baseline relative abundance of *Bacteroides* (Spearman p=0.018).

To investigate microbial genera related to reduced LDL-C, we used EXPLANA, a software workflow for exploratory analysis with mixed effects random forest (MERF). ^52^ While inputting 490 bacterial genera, HIV-infection status, MSM status, and timepoint as predictors of LDL-C measurements over the three timepoints, we found that could predict LDL-C values that explained 9.2% of the variation, with 12 features selected as significantly important using the Boruta method (Fig. S5A left panel 1). ^53^ We also used EXPLANA to calculate deltas between all pairs of timepoints in both the outcome (LDL-C) and predictors prior to application of a MERF. When analyzing pairwise changes, a 19.1% variation in LDL-C delta values was explained and 10 features were selected as important (Fig. S5A right panel Fig. S5B). When the same feature selection method was performed using the WD cohort, the microbiome did not show any strong predictive capabilities for LDL-C. *Collinsella* and *Negativibacillus* were identified as important in both models (Fig. S5A). Decreased *Collinsella* and increased *Bilophila* were related to negative changes in LDL-C (Fig. S5C).

### Biopsy Profiling Revealed Changes in Host Transcription:

Similar exploratory analyses assessing changes in the microbiome, immune cells, and host transcription were performed on 54 colonic biopsy samples collected by flexible sigmoidoscopy. Differences were observed in mucosal microbiome by HIV status but not MSM status (Fig. S6A). No significant changes with AD in the mucosal microbiome were observed (Fig. S6B and C). In LMEM (Fig. S7A), immune cells did not show any strong relationship with time on the AD, whereas WD was associated with elevated CD3+ CD56+ NK T Cells (Fig. S7B). Biopsy immune cells showed no relationship with LDL-C (Fig. S7D).

**Figure 5:**
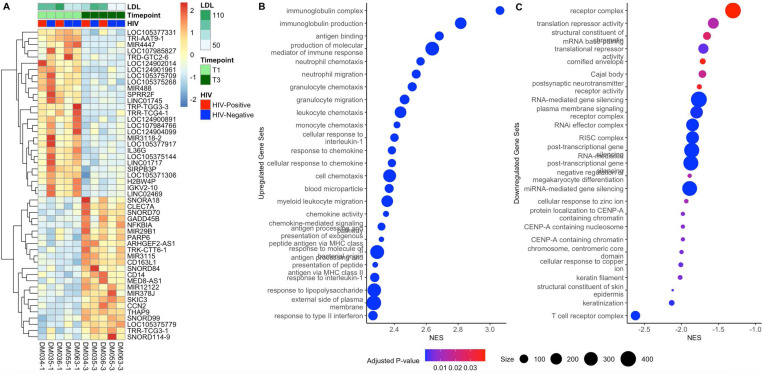
Biopsy Transcriptome Differences by HIV and Time on AD. (A) Heatmap of expression of 50 most differentially expressed genes from RNA-sequencing identified by Limma with transcripts clustered according to Euclidean distance. Samples are shown in columns, and the corresponding metadata is shown above the heatmap. (B) 24 most enriched gene sets, and (C) 24 most depleted gene sets. Higher normalized enrichment scores (NES) indicate enrichment at the later timepoint. LDL=low density lipoprotein cholesterol, HIV=human immunodeficiency virus, NES=normalized enrichment score.

In a subset of 5 MSM individuals on the AD, RNA-sequencing was performed on biopsy samples collected pre- and post-diet intervention to characterize host gene expression. Limma was used to identify differential expression between T1 and T3.^54^ Fig. 5A shows a heatmap of expression of the 50 most differentially expressed genes. Although only one individual gene was significant after FDR correction (decrease in a tRNA with the AD), gene set enrichment analysis revealed increased expression of genes in immune activation/inflammation related pathways and decreased expression of genes involved in many signaling and regulation-related gene sets (Fig. 5B and 5C).

## Discussion:

The impact of a short-term AD in HIV(+)MSM and controls was assessed in a controlled clinical trial. By quantifying a wide range of outcomes, the health impacts of an AD on this at-risk population were comprehensively characterized. High-fat diets have been found to exacerbate SIV immune activation and pathogenesis.^36^ Conversely, high-fiber, low-fat diets have been associated with reduced inflammation and improved metabolic outcomes.^55–58^ The PRBP microbiomes of MSM at risk for HIV infection from sexual behaviors are compositionally similar to those from agrarian populations, and we had thus hypothesized that they may have particularly strong health benefits from an AD intervention.^34,59^ Indeed, we found that high *Prevotella* and low *Bacteroides* at baseline predicted greater reductions in LDL-C with the AD. This result is consistent with a prior study showing that individuals with more PRBP microbiomes exhibited better glucose tolerance response following a short term high-fiber intervention, and suggests that the AD may be beneficial for the metabolic health of individuals with this microbiome type.^35^

A step-wise intervention of provided meals followed by guided instruction and home preparation allowed for a longer intervention at reduced expense. While self-feeding resulted in variable compliance, a significant shift towards AD macronutrient targets was maintained, with those macronutrients also being reported to allow for future generalizability. Including a WD control also arm allowed us to identify effects driven by the intervention itself not specific to the AD. Expected dietary changes were observed with the AD, while subtle changes in dietary macronutrient content were detected in the WD compared to baseline. This is to be expected because, although the WD was based on American norms, there is individual variation. Meals were prepared using hygienic conditions, limiting exposure to pathogens. This is suspected to be a driving factor of the decreased LBP between T1 and T2 in HIV(+)MSM on the WD. Metabolic markers HDL-C and LDL-C were only responsive to the AD intervention, particularly in HIV(+)MSM. The decrease in LDL-C with the AD suggests the benefits of AD for decreasing the incidence of CVD and diabetes.^60,61^ Indeed, the median reductions in LDL-C of 0.4138 mmoL/L at T2 and 0.2845 mmol/L at T3 has been associated with a 11.6% (T2) and 8.0% (T3) reduced risk of CVD.^62^ Although HOMA-IR and triglycerides did not change with the AD, this could be due to limited representation of individuals with high baseline levels, which was associated with less reduction.

Decreased HDL-C observed with the AD may be concerning because HDL-C is generally thought to have beneficial health effects.^63^ HDL-C has previously been shown to drop in similar interventions.^64–66^ In our intervention, reduced levels of HDL-C were not sustained at T3, and did not decrease in participants with low baseline levels. Also, HDL pools can differ in function, including whether they are pro- or anti-inflammatory and their capacity to perform cholesterol efflux; and thus LDL is a more accurate predictor of CVD.^67^ Furthermore, HIV infection is associated with impaired HDL antioxidant function, complicating its role in the study population.^67^ The plasma inflammatory markers did not significantly change with the AD, except when restricting the analyses to only the individuals with higher baseline values. This suggests that the AD could be beneficial specifically to those with higher levels of inflammation. While a previous study showed increased inflammatory measures in SIV-infected macaques fed a high-fat diet, that study only explored untreated infection, where inflammation would be more consistently elevated compared to this study population on effective ART. ^36^

Those on the AD had decreased blood immune cell populations, including PD-1+ and CD40+ T cells and total monocytes as well as differential classical and intermediate subtype monocytes. Reduced T cells expressing the exhaustion marker PD-1 from individuals on AD, which is consistent with past studies, is relevant, since PD-1 expression in T cells is positively associated with HIV replication, suggesting a potential dietary impact on residual viral replication.^68–73^ Regarding CD40, a high cholesterol diet has also been shown to increase CD40 immunopositivity in developing atherosclerotic plaques in a rabbit model.^74^ CD40 has also been shown to play a pathogenic role in and atherosclerosis onset.^75,76^ These findings suggest AD could be conferring beneficial metabolic effects by reducing CD40+ T cells. The reported diet-induced changes in monocytes is not surprising, as fasting reduces circulating monocytes, particularly CD14+CD16+ intermediate monocytes, and reduced peripheral monocyte number has been linked to dietary protein and glucose.^77^ Further, because monocytes participate in the progression of atherosclerotic lesions and CD14+CD16+ intermediate monocytes have been associated with heart disease, these AD-associated changes may be clinically relevant.^78–80^

No AD-associated changes in immune cell populations or microbes were observed in colonic biopsy, possibly due to limited sample number. However, RNA-Sequencing analysis revealed an increase in expression of host genes related to immune activation. AD was associated with reduced expression of genes associated with regulatory mechanisms and differential expression of several non-coding RNAs. Diet, particularly fat intake, has been previously shown to impact miRNA regulation, with implications for lipid metabolism. ^81,82^ Further, tRNAs and other non-coding RNAs have been associated with altered host lipid metabolism.^83,84^ Although this study’s small sample size is limiting, these data do suggest the unexpected finding that the AD actually increased immune activation at the mucosal interface.

In this study population, MSM differed from MSW by having relatively PRBP gut microbiomes, as has been previously reported.^23,24^ Since a PRBP gut microbiome has been associated with ADs, it is not surprising that pronounced changes in the gut microbiome with the AD intervention did not occur, as individuals perhaps already had a gut microbiome suited to AD. ^32,33,85^ Results describing microbiome changes in *Prevotella* and *Bacteroides* from past interventions have been mixed. One study reported *Prevotella* to increase from short-term fiber dietary interventions, whereas another supported the stability of microbial *Prevotella* and *Bacteroides* from short-term changes in fiber and fat.^33,35^ However, baseline microbiome characteristics predicting dietary response are consistent with past studies.^49^ In fecal samples, while diet had no relationship with changes in the microbiome, it appeared MSW had relatively stable microbiomes compared to MSM groups. Drivers of this heightened variability are unknown but could include sexual behaviors that increase exposures to different microbiomes or inherent attributes of PRBP microbiomes. While AD-associated microbial changes did not occur, exploratory analysis suggested that changes in particular microbes had some predictive power for changes in LDL-C with the AD, which could serve as targets for future studies.

The PRBP microbiome of MSM has previously been associated with higher levels of systemic immune activation and induction of higher gut immune activation in gnotobiotic mice, suggesting negative health effects. ^86^ Our results instead suggest a positive health benefit, whereby increased diversity and altered composition of commensal gut microbes confer a greater capacity to benefit metabolically from an AD.^87,88^ A diverse agrarian type microbiome in MSM has been linked with sexual behaviors that may increase exposures to commensal and pathogenic bacteria, such as having a high number of different sexual partners.^34^ Increased exposures to diverse microbes through sexual behaviors in high-risk MSM may not only increase susceptibility to infectious disease, but also to beneficial commensals.

Taken together, this study identified beneficial effects of a short-term AD intervention relevant to PLWH. By reducing LDL-C levels, lowering immune cell activation and T-cell exhaustion, and conferring a reduction in IL-6 in participants with elevated inflammation, the AD could serve as a powerful tool in reducing metabolic and immune dysfunction in PLWH. Further, the response of PRBP microbiomes to AD and AD-associated changes in mucosal host transcription could serve as interesting preliminary findings for future work.

## Online Methods:

### Study Enrollment:

Participants were residents of the Denver, Colorado, USA metropolitan area, and were recruited and studied at the Clinical Translational Research Center (CTRC) of the University of Colorado Anschutz. Recruitment took place between February of 2016 and January of 2020. HIV(+)MSM had HIV-1 infection defined as a positive antibody test or plasma HIV-1 RNA, were treated with ART (minimum of three antiretroviral drugs in regimen) for at least 12 months with no changes in antiretroviral drugs over the past 6 months and had plasma HIV-1 RNA ≤ 50 copies/mL for the preceding six months. HIV(−) participants had a negative third generation HIV antibody/antigen test at screening. MSM status was based on a sexual behavior questionnaire. Entry criteria for all groups included age 18 to 65 years, a body mass index between 21–29 kg/m^2^ (non-obese), and stable weight for at least 3 months (≤15% change in body weight). Participants were excluded if they had used systemic antibiotics within the prior two months, had active chronic infection such as hepatitis B or C or an active malignancy that required systemic chemotherapy, or had diabetes. Informed consent was obtained from all participants and the study was approved by the Colorado Multiple Institutional Review Board (CoMIRB: 15–1692). Additional details are available at clinicaltrials.gov NCT02610374.

### Study Design and Randomization:

The trial was randomized and unblinded. After enrollment, participants were randomly assigned, using an excel random number generator, with equal probability to the AD (indicated by a 1) or the AD (indicated by a 2). The random allocation sequence was maintained by a research assistant. The initial primary outcome selected was plasma IL-6 (See Immune Data Collection Section), which was used to quantify AD-associated reductions in systemic inflammation. The target sample set included 50 HIV(+)MSM, 24 HIV(−)MSM, and 24 HIV(−)MSW, with a target 1:1 allocation to WD and WD. This was determined because previous work found that a reduction of 0.08 pg/mL in plasma IL-6 is associated with a 16% change in mortality risk, indicating a change of 0.08 pg/mL is clinically relevant.^89^ The target sample set included 50 HIV(+)MSM, 24 HIV(−)MSM, and 24 HIV(−)MSW, with a target 1:1 allocation to WD and WD. This was determined because previous work found that a reduction of 0.08 pg/mL in plasma IL-6 is associated with a 16% change in mortality risk, indicating a change of 0.08 pg/mL or over is clinically relevant.^89^ Based on previously reported IQR of PLWH on effective antiretroviral therapy (median 1.89 IQR (1.15–3.42) pg/mL), assuming a correlation of 0.7 between baseline and post-intervention, 25 HIV(+)MSM per diet arm would allow for the detection of a difference of 0.4 pg/mL with 88% power.^90^ Based on previously reported IQR of HIV-negative individuals (median 1.29 IQR (0.80–2.07) pg/mL), 12 HIV(−)MSM and 12 HIV(−)MSW per diet arm would allow for the detection of a difference of 0.25 pg/mL with 80% power.^90^ The calculations for sample size were conducted using the software package G*Power.^91^

### Diet Intervention:

After randomization participants completed a 4-week diet intervention. Baseline diets were surveyed using the Diet History Questionnaire II (DHQ2) of the National Cancer Institute.^92^ Macronutrient totals were estimated based on the survey using the Diet*Calc Analysis Program version 1.5.0.^93^ For the first two weeks of the diet intervention, all food was prepared and supplied by the Colorado Clinical and Translational Sciences Institute (CCTSI) Nutrition Core at the University of Colorado, Anschutz Medical Campus. The total energy intake for each individual was determined using the Mifflin-St. Jeor equation to estimate Resting Metabolic Rate (RMR) with an activity factor of 1.4 to account for daily physical activity.^94,95^ The CCTSI Nutrition Core provided counseling and menus during the second two weeks for individuals to self-prepare meals. Individuals completed a 24-hour food recall questionnaire per week of self-prepared meals, which was analyzed using the Nutrition Data System for Research software version 2017 developed by the Nutrition Coordinating Center (NCC), University of Minnesota, Minneapolis, MN. Averages from both questionnaires were obtained. Dietary values were obtained using three metrics as described, per individual, for baseline, the first two weeks, and the second two weeks and used with PCA to assess changes in diet over time.

To avoid negative medical consequences on AD, instead of providing lower protein, high protein foods commonly consumed in Africa were provided, such as lean whole meats and eggs. Foods high in resistant starch (RS) and yams were used to mimic cold maize porridge and cassava, agrarian African staples.^96^ Thus, the intervention diet was similar in composition to the agrarian African diet in having a high complex-to-simple carbohydrate ratio, but palatable to this study population.^97–99^ Study stopping guidelines included any serious adverse event occurring during or preceding the research mucosal biopsy procedure.

### Fecal, Blood, and Biopsy Collection:

Fecal samples were collected at T1, T2 and T3. Study participants collected a fecal sample using a commode specimen collector. Fecal samples were stored at −4°C during transport (for <48 hours) via UPS, aliquoted and transferred to −80°C upon delivery to the lab.

A colonic biopsy was collected at T1 and T3 on 54 participants. Participants were given two Fleet saline enemas (Prestige Consumer Healthcare, Inc., Lynchburg, VA, USA) followed by flexible sigmoidoscopy with collection of 30 pinch biopsies from the colorectal tissue using 2.4mm forceps. Four pinch biopsies were put into 0.25 mL of RNALater (Thermo Fisher Scientific, Waltham, MA, USA) for microbiome (16S rRNA) analysis. The remaining biopsies were put in 10 mL low barium PBS for use with Cytometry by Time of Flight (CyTOF). The biopsies were digested for 1.5 hours with collagenase (1 mg/mL) and DNAse (5 ul/mL) as previously described.^100^ Pinches were then mashed on and filtered through a 70-micron nylon cell strainer and washed with 15 mL low barium PBS, centrifuged, and resuspended in 2 mL low barium PBS. Equal amounts of cells were divided and immediately stained for CyTOF.

Whole blood samples were collected in sodium heparin vacutainers. For plasma extraction, samples were centrifuged at 1,700 rpm for 10 minutes. Plasma was then aliquoted and stored at −80°C.

### Immune Data Collection:

#### ELISAs:

Plasma inflammatory markers, including lipopolysaccharide binding protein (LBP) (HycultBiotech Wayne, PA, USA, C-reactive protein (CRP), and interleukin-6 (IL-6), were measured using enzyme-linked immunosorbent assays (ELISAs) (R&D Systems Minneapolis, MN, USA).

#### CyTOF:

Because cell death was observed upon freezing with the limited amount of biopsy tissue, samples were processed and run fresh within 3 hours of sample collection. PBMC and pinch biopsy single cell suspensions were stained for live–dead cell distinction using 2.5 μM Cisplatin (Fluidigm, South San Francisco, CA, USA). Cells were re-suspended in 65 μl barium free FACS buffer (low barium PBS with 0.1% BSA and 2 mM EDTA) and incubated for 30 min at 4°C with a 35 μl cocktail of metal-conjugated antibodies (1 μl each) (Table S1). Cells were washed and resuspended with MaxPar fix with DNA intercalator (0.125 μM Iridium-191/193; Fluidigm, South San Francisco, CA, USA) and EQ Four Element Calibration Beads (Fluidigm, South San Francisco, CA, USA) were added. Cells were acquired using a CyTOF2 mass cytometer (Fluidigm, South San Francisco, CA, USA), CyTOF software v.6.0.626 with noise reduction, a lower convolution threshold of 200, event length limits of 10–150 pushes, a sigma value of 3, and a flow rate of 0.045 ml/min. Runs were concatenated using the FCS file concatenation tool from Cytobank and normalized using the EQ Four calibration beads. The blinded manual gating strategy used high confidence immune populations that did not express the marker of interest or that had definitive bimodal staining pattern to define marker positivity of other immune cell subsets based on careful, blinded manual gating. These gates were then directly applied to the population of interest. A representative gating strategy on blood immune populations is shown in Fig. S3.

### Metabolic Data Collection:

Whole blood samples were collected by phlebotomy at T1, T2, and T3. Samples were sent to the Colorado Translational Research Center (CTRC) for measurements of HDL-C, LDL-C, TG, glucose, and insulin. HOMA-IR was calculated using fasting glucose and insulin levels as described by Matthews et al. ^101^ To be consistent with previously published thresholds,^42–44^ the definition of healthy serum lipids included HDL-C concentrations over 40 mg/dL in men and 50 mg/dL in women, LDL-C concentrations below 100 mg/dL, and Triglyceride concentrations below 100 mg/dL. Additionally, healthy HOMA-IR was defined as below 2.5.^102^

### 16S Sequencing:

DNA was extracted from fecal and biopsy samples using a Qiagen standard DNeasy PowerSoil Kit per the manufacturer’s instructions. Extraction of the DNA was followed by PCR amplification using barcoded primers designed to target the V4 variable region of the 16S rRNA gene. Experiments were performed in accordance with the Earth Microbiome Project 16S Illumina Amplicon standard protocols for 515F:806R primer constructs.^103^ Invitrogen PicoGreen was used to quantify each PCR product. Equal amounts of DNA were pooled together, and the cleaning was done with the MoBio UltraClean PCR Clean-up Kit.

### Microbiome Analysis:

QIIME 2 version 2022.8.0 was used to conduct microbiome analysis of the 16S sequencing data.^51^ As previously described, raw sequences were demultiplexed and denoised using the DADA2 q2 plugin, as previously described.^104^ During DADA2 processing, all sequences were truncated to 230 bp. Sequences were binned into 99% ID operational taxonomic units (OTUs). OTUs were classified using QIIME 2 with classifiers pretrained on the Greengenes2 database, and OTUs not classified at the phylum level, features classified as mitochondria or chloroplasts and features present in less than 20% of samples were excluded from analysis.^105^ Samples were rarefied to 2790 and 12,922 reads, for biopsy and feces, respectively, and relative abundances were calculated by normalizing to the total number of reads. A rooted phylogenetic tree was generated in QIIME 2 using the SEPP plugin, which allowed for the measurement of Shannon Diversity, Faith’s Phylogenetic Diversity, as well as weighted and unweighted UniFrac distances.^106^

### RNA Sequencing:

RNA was extracted using the Qiagen AllPrep kit followed by ribo-depletion with QIAGEN FastSelect. Ribo-depleted RNA was sequenced using the Illumina Novaseq platform at the CU Genomics Core. Raw reads were trimmed using Trimmomatic under standard parameters and quality checked using FastQC and MultiQC. Sequenced RNA was aligned using HISAT2 to the GRCh38 human reference genome, and transcript count tables were generated using featureCounts.^107,108^

### Statistical Analyses:

Wilcoxon Ranked-based tests and Kruskal-Wallace tests were used for cross sectional comparisons across diets and study groups, respectively. However, for microbiome data, ANCOM-BC was used to compare pairwise between groups, to account for the compositional nature of the data.^47^ Longitudinally, for stratified analyses in subgroups, Friedmans tests were used. When assessing longitudinal changes in immune cells, metabolic markers, and inflammatory markers in all groups stratified by diet, MSM and HIV status were incorporated into LMEMs. However, ANCOM-BC was used to assess longitudinal changes in the microbiome data. Additionally, Adonis statistical tests were used to assess differences in overall microbiome composition by MSM status, HIV status, and Diet. For RNA-sequencing, Limma was used to identify cross sectional differences and longitudinal changes.^54^ Gene set enrichment analysis was performed with the clusterProfiler R package.^109^

### Exploratory Analysis:

EXPLANA was used for exploratory analysis to identify features related to LDL-C. A random effect for individuals was used to adjust for non-independence (repeated measurements).^52^ There were 500 trees used per model with a max feature fraction of 0.75 of the input features for each split per decision tree in the forest. 10 iterations were performed for mixed-effects Random Forests. BorutaSHAP was used to find features that repeatedly performed better than shuffled versions of all input features. Features were considered important if they performed better than 100% of the SHAP importance score of the best shuffled feature using 100 trials, p=0.05. Categorical variables were binary encoded.

## Supplementary Material

Supplement 1

## Figures and Tables

**Table 1:** Study Population: Baseline data split by HIV and MSM status with data presented as n, median (interquartile range) or n (%), unless otherwise stated. HIV=human immunodeficiency virus, MSM=men who have sex with men, MSW=men who have sex with women, BMI=body mass index, IQR=inter-quartile range, LDL-C=low-density lipoprotein cholesterol, HDL-C=high-density lipoprotein cholesterol, HOMA-IR=Homeostatic Model Assessment for Insulin Resistance, LBP=lipopolysaccharide binding protein, IL-6=Interleukin-6, CRP=C reactive protein, NRTI=nucleoside reverse transcriptase inhibitor, INSTI=integrase strand transfer inhibitor, NNRTI=non-nucleoside reverse transcriptase inhibitor.

Cohort	HIV(+)MSM	HIV(−)MSM	HIV(−)MSW
Sample Number	36	21	9
Age (Median (IQR))	46.03 (33.11–54.57)	39.57 (30.24–46.96)	29.38 (24.07–34.57)
BMI (Median (IQR))	25.90 (23.30–28.40)	25.60 (24.00–27.00)	26.30 (25.60–27.00)
**Diet**			
Agrarian (Count (%))	18 (50.0%)	13 (61.9%)	4 (44.4%)
Western (Count (%))	18 (50.0%)	8 (38.1%)	5 (55.6%)
Distance to Standard Agrarian (Median (IQR))	1.63 (1.52–1.87)	1.68 (1.37–1.8)	1.59 (1.51–1.69)
**Metabolic Markers**			
HDL-C (mg/dL) (Median (IQR))	40.00 (34.50–52.00)	49.00 (39.00–55.00)	42.00 (34.00–48.00)
LDL-C (mg/dL) (Median (IQR))	102.00 (83.00–117.50)	94.00 (78.00–121.00)	80.00 (72.00–112.00)
Triglycerides (mg/dL) (Median (IQR))	119.00 (93.00–203.00)	83.00 (63.00–181.00)	99.00 (84.00–130.00)
HOMA-IR (Median (IQR))	1.44 (0.78–2.32)	0.92 (0.76–2.53)	1.06 (0.64–1.17)
**Inflammatory Markers**			
LBP (μg/μL) (Median (IQR))	34.96 (33.03–36.54)	35.6 (33.73–36.64)	35.03 (32.03–35.58)
IL-6 (pg/mL)(Median (IQR))	3.95 (1.91–5.50)	2.81 (1.73–3.41)	2.75 (2.32–8.02)
CRP (mg/L)(Median (IQR))	3.21 (2.3–5.22)	2.68 (1.95–4.07)	2.39 (1.22–3.42)
**Alpha Diversity**			
Fecal Shannon (Median (IQR))	5.54 (5.05–5.83)	5.95 (5.60–6.45)	5.41 (5.12–5.83)
Fecal Faith (Median (IQR))	18.91 (15.58–21.89)	22.33 (17.74–26.90)	21.78 (19.00–26.07)
Biopsy Shannon (Median (IQR))	5.44 (4.69–5.70)	5.86 (5.37–6.16)	5.72 (5.63–5.85)
Biopsy Faith (Median (IQR))	23.94 (15.26–33.22)	19.48 (14.31–22.37)	26.82 (14.90–35.39)
**T Cell Percentages**			
CD4+ T cells/mL (Median (IQR))	628 (514.50–945.25)	NA	NA
CD8+ T cells/mL (Median (IQR))	709 (590.00–925.50)	NA	NA
**Antiretroviral Therapy Combinations (N=34)**			
2 NRTIs and INSTI	23 (67.6%)	NA	NA
2 NRTIs and NNRTI	6 (17.6 %)	NA	NA
Other	5 (14.7%)	NA	NA

**Table 2:** Standard Diet Compositions: Average values of dietary components at the second timepoint when participants were receiving prepared meals

Diet Composition	Agrarian Target/Controlled Feeding	Agrarian Free-Living Intake	Western Target/Controlled Feeding	Western Free-Living Intake
Fat (%)	15 ± 0.0002	21.3 ± 1. 2	30 ± 0.0002	38.6 ± 0.8
Saturated Fat (%)	3.2 ± 0.0002	3.5 ± 0.0002	10 ± 0.0009	10 ± 0.002
Carbohydrate (%)	70 ± 0.0006	55.4 ± 1.2	55 ± 0.0003	45.9 ± 0.9
Protein (%)	15 ± 0.0002	21.9 ± 0.8	15 ± 0.00017	15.4 ± 2.9
Sodium (mg/day)	1500 ± 9.2	1053 ± 128	3500 ± 12.75	3985 ± 199
Fiber (g/1000 kcal)	22 ± 0.1	21.9 ± 0.8	9 ± 0.07	6.3 ± 0.3
Sugar (g/1000 kcal)	30 ± 0.1	27.1 ± 2.1	57 ± 0.2	47.5 ± 1.9
