# Supplementary material for: Agrarian Diet Improves Metabolic Health in HIV-positive Men with Prevotella-Rich Microbiomes: Results from a Randomized Trial
Source: Res Sq. 2024 Nov 15:rs.3.rs-5349309. Preprint. [Version 1] doi: 10.21203/rs.3.rs-5349309/v1 (PMC11601827; doi:10.21203/rs.3.rs-5349309/v1)
Supplement: Supplement 1 [file NIHPPRS5349309V1-supplement-1.pdf]

Supplement:

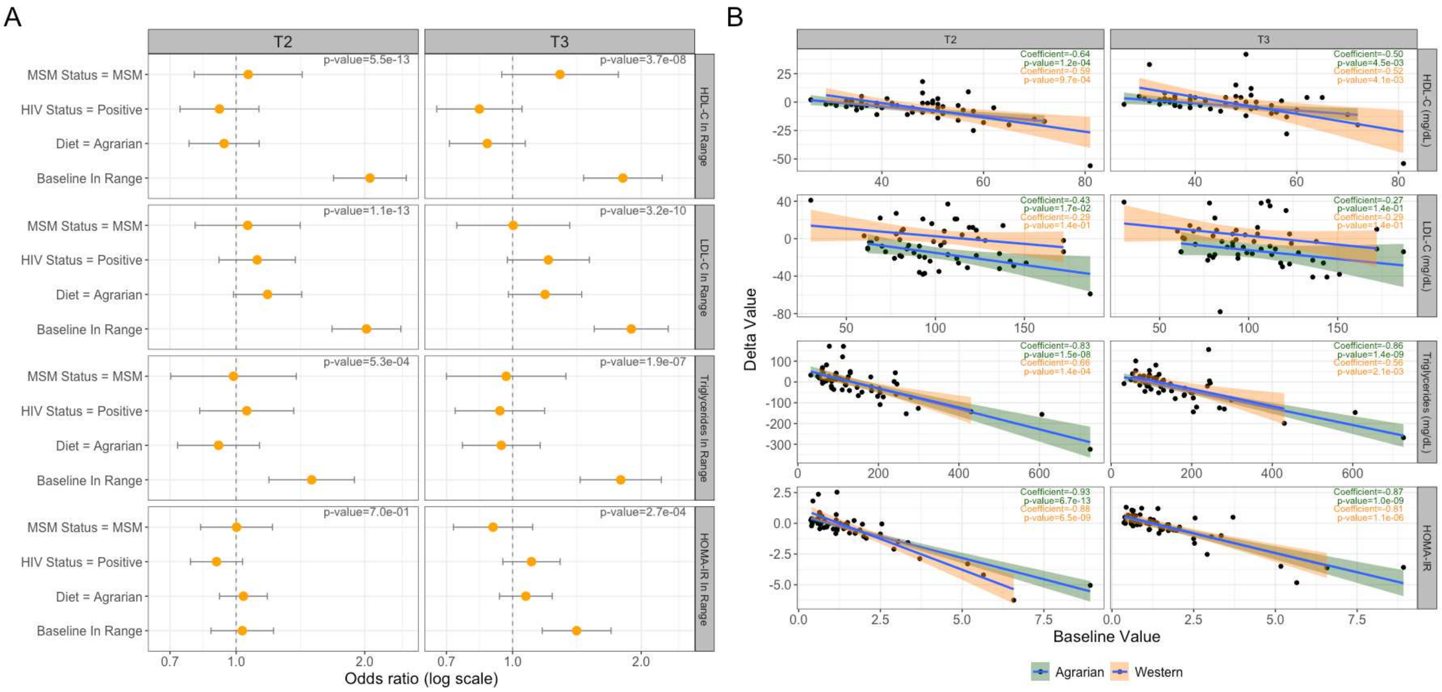

Figure S1: Baseline Metabolic Measures Predictive of Measures at Later Timepoints. (A) Results of logistic regressions of metabolic measures being in the healthy range of metabolic markers (row panels) at different timepoints (column panels), y-axis represents predictive variables, x-axis represents odds ratios, brackets indicate 95% confidence intervals, and intercept p-values are in the top right corners; (B) Scatter plots of baseline values of different metabolic measures (row panels) to changes in those values at different timepoints (column panels), x-axis is represent baseline values, y-axis represents the change from T1 to T2 (left) and T1 to T3 (right); Spearman correlation coefficients and p-values are displayed in the top right corner. MSM=men who have sex with men, HIV=human immunodeficiency virus, HDL-C=high-density lipoprotein cholesterol, LDL-C=low-density lipoprotein cholesterol, HOMA-IR=Homeostatic Model Assessment for Insulin Resistance.

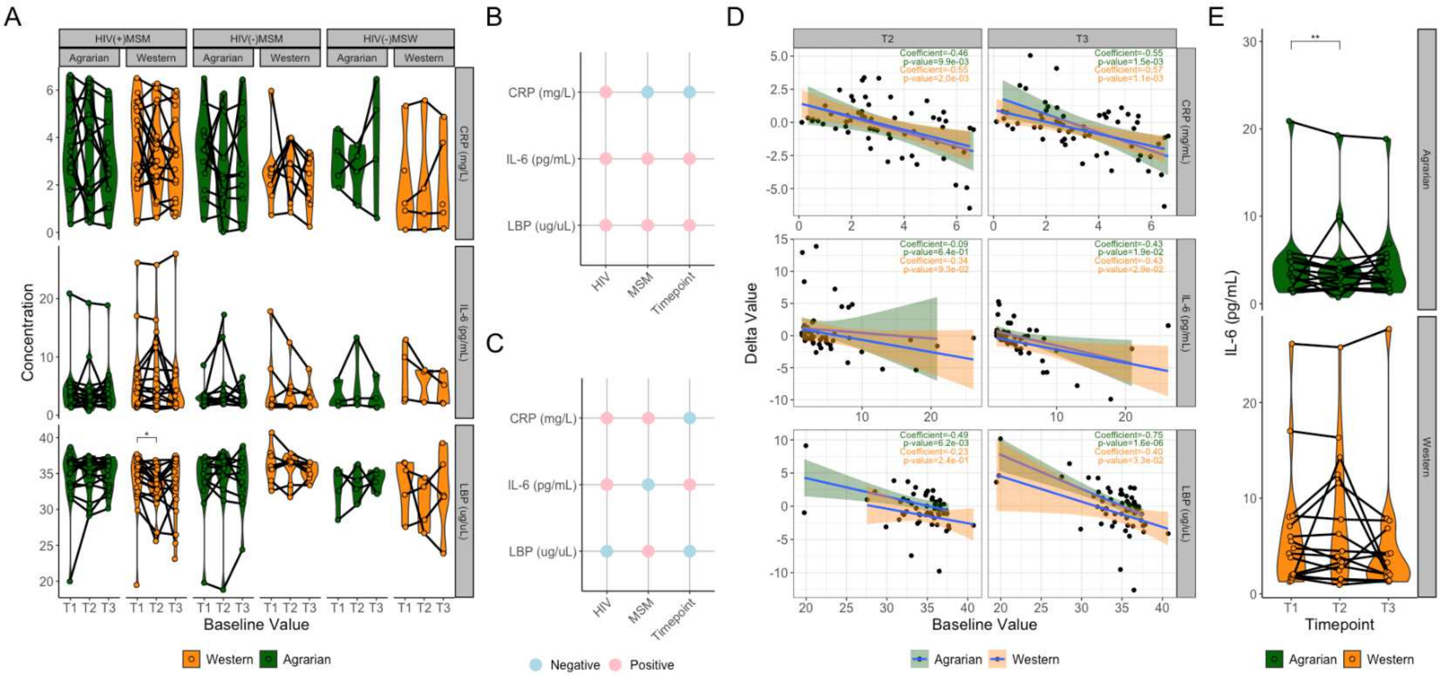

Figure S2: Baseline Values of Inflammatory Markers and LBP Predictive of Measures at Later Timepoints. (A) Violin plots overlaid with spaghetti plots of CRP, IL-6, and LBP colored by diet with brackets indicating significance as determined by Friedman tests with Bonferroni multiple comparisons correction; *-p≤0.05, **-p≤0.01, ***-p≤0.001, ****-p≤0.0001. **.** (B and C) Coefficients of linear mixed-effects models (LMEMs) relating inflammatory markers to MSM status, HIV-infection status, and timepoint in (B) those on the agrarian diet and (C) those on the western diet. Red indicates a positive relationship with positive HIV-infection status, MSM status, and change between T1 and T3 with the diet intervention, while blue indicates the opposite. Dark red and blue in panel A represent significance at p<0.05. P-values determined by analysis of variance (ANOVA) of full LMEM model (Immune Cell Population ~ HIV+MSM+(1|StudyID) vs model removing predictor of interest. Line (D) Scatter plots of baseline values of different immune measures (row panels) to changes in those values at different timepoints (column panels), x-axis represents baseline values, y-axis represents the change from T1 to T2 (left) and T1 to T3 (right); Spearman correlation coefficients and p-values are displayed in the top right corner (E) Violin plots of IL-6 in subjects starting with values in the top 50th percent split and colored by diet brackets indicating significance as determined by Friedman tests with Bonferroni multiple comparisons correction; *-p≤0.05, **-p≤0.01, ***-p≤0.001, ****-p≤0.0001. HIV=human immunodeficiency virus, MSM=men who have sex with men, MSW=men who have sex with women, CRP=C-reactive protein, IL-6=interleukin 6, LBP=lipopolysaccharide binding protein.

Target	General Phenotype	Metal	Clone	Catalog
CD3	T cells	170Er	UCHT1	3170001B
CD4	Helper T cells	176Yb	RPA-T4	3176010B
CD8a	Cytotoxic T cells	146Nd	RPA-T8	3146001B
CD25 (IL-2R)	Treg	149Sm	2A3	3149010B
CD27	B and T cell maturation	167Er	O323	3167002B
CD38	T cell activation	172Yb	HIT2	3172007B
CD45	Leukocytes	141Pr	HI30	3141009B
CD45RA	T cell maturation	169Tm	HI100	316900B
CD69	T cell activation	144Nd	FN50	3144018B
CD90 (Thy-1)	T cell activation/Th17	161Dy	5e10	3161009B
CD103	Mucosal Homing	151Eu	Ber-ACT8	3151001B
CD127 (IL-7R)	T cell activation/Tregs	168Er	A019D5	3168017B
CD132	Activation	166Er	TUGh4	Custom
CD152 (CTLA-4)	Exhaustion/Tregs	173Yb	14D3	Custom
CD161	gd T cells/MAIT	159Tb	HP-3G10	3159004B
CD183 (CXCR3)	Th1	163Dy	G025H7	3163004B
CD185 (CXCR5)	TFH cells	164Dy	51505	3163004B
CD195 (CCR5)	Co-receptor for HIV	156Gd	NP-6G4	3156015A
CD279 (PD-1)	T cell exhaustion	175Lu	EH12.2H7	3175008B
FoxP3	Tregs	162Dy	259D/C7	3162024A
Va7.2	Maits	153Eu	3C10	3153024B
CD1c	Gut DCs	152Sm	L161	Custom
CD11b (Mac-1)	Macrophage	209Bi	ICRF44	3209003B
CD11c	Dendritic Cells	147Sm	Bu15	3147008B
CD14	Monocytes	160Gd	M5E2	3160001B
CD16	Nks and monocytes	145Nd	3G8	3145008B
CD19	B cell	142Nd	HIB19	3142001B
CD40	mDC activation	165Ho	5C3	3165005B
CD56	NK	155Gd	B159	3155008B
CD68	Macrophages	171Yb	Y1/82A	3171011B
CD86	APC Activation	150Nd	IT2.2	3150020B
CD123 (IL-3R)	Dendritic Cells	143Nd	6H6	3143014B
CD163	Monocytes/Macrophages	154Sm	GHI/61	3154007B
HLA-DR	APC/T cell Activation	174Yb	L243	3174001B
CD274 (PD-L1)	APC activation/exhaustion	148Nd	29E.2A3	3148017B

Table S1: CyTOF mAb Panel: Target antibody panel used to characterize immune populations in blood and colonic biopsy in Cytometry by Time-of-flight mass spectrometry (CyTOF). Treg=regulatory T cell, gd=gamma-delta, MAIT=mucosal-associated invariant T, HIV=human immunodeficiency virus, DC=dendritic cell, mDC=myeloid dendritic cell, NK=natural killer cell, APC=antigen presenting cell.

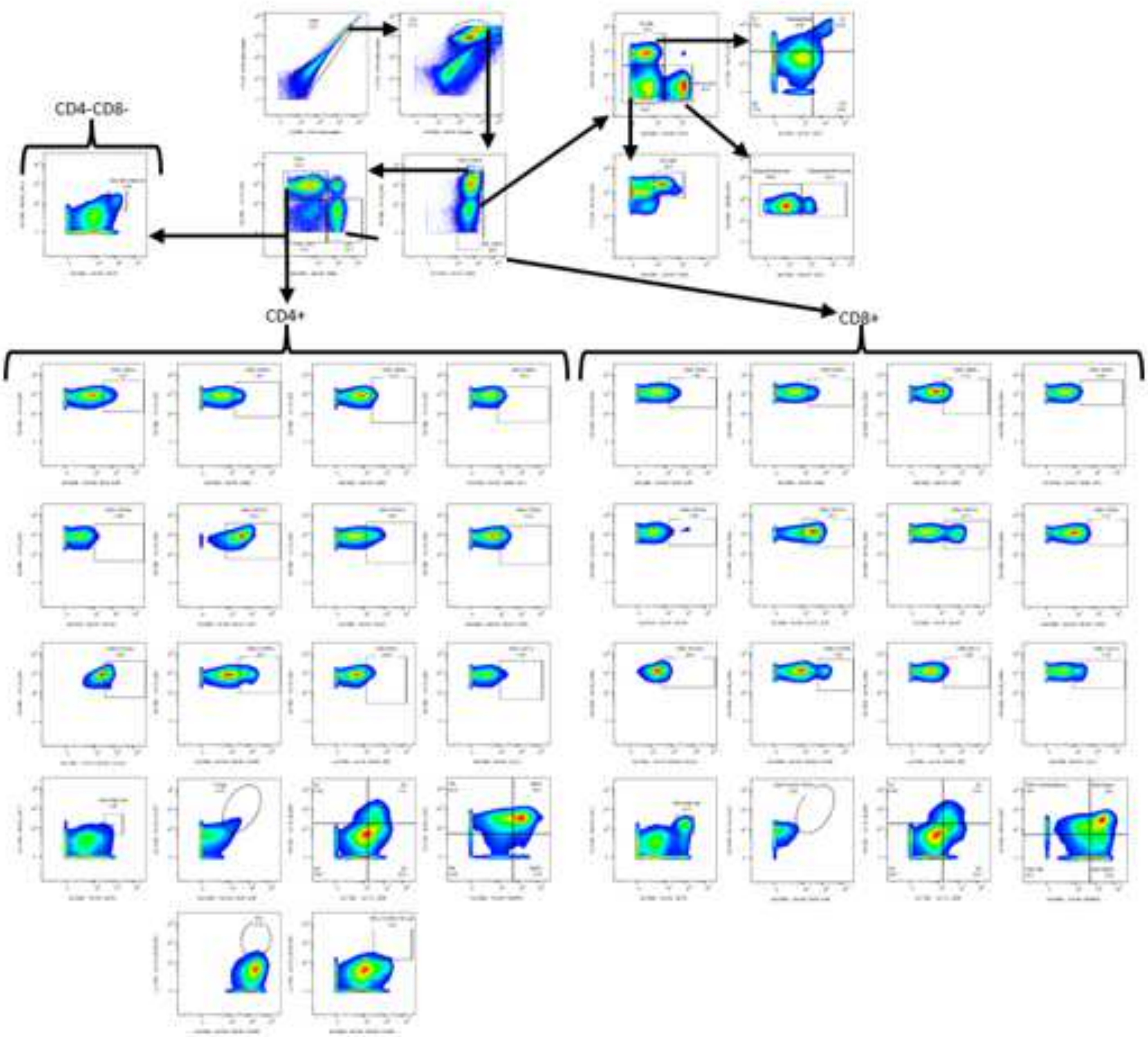

Figure S3: Representative CyTOF Gating Hierarchy. Visual representation of cytometry by time-of-flight mass (CyTOF) and gating strategy from a peripheral blood mononuclear cell (PBMC) sample showing the identification of key immune cell populations. Intact viable cells were selected followed by manual gating of major immune cell subsets including CD4+ T cells, CD8+ T cells, monocytes and B cells with T cell populations being further evaluated for additional subsets.

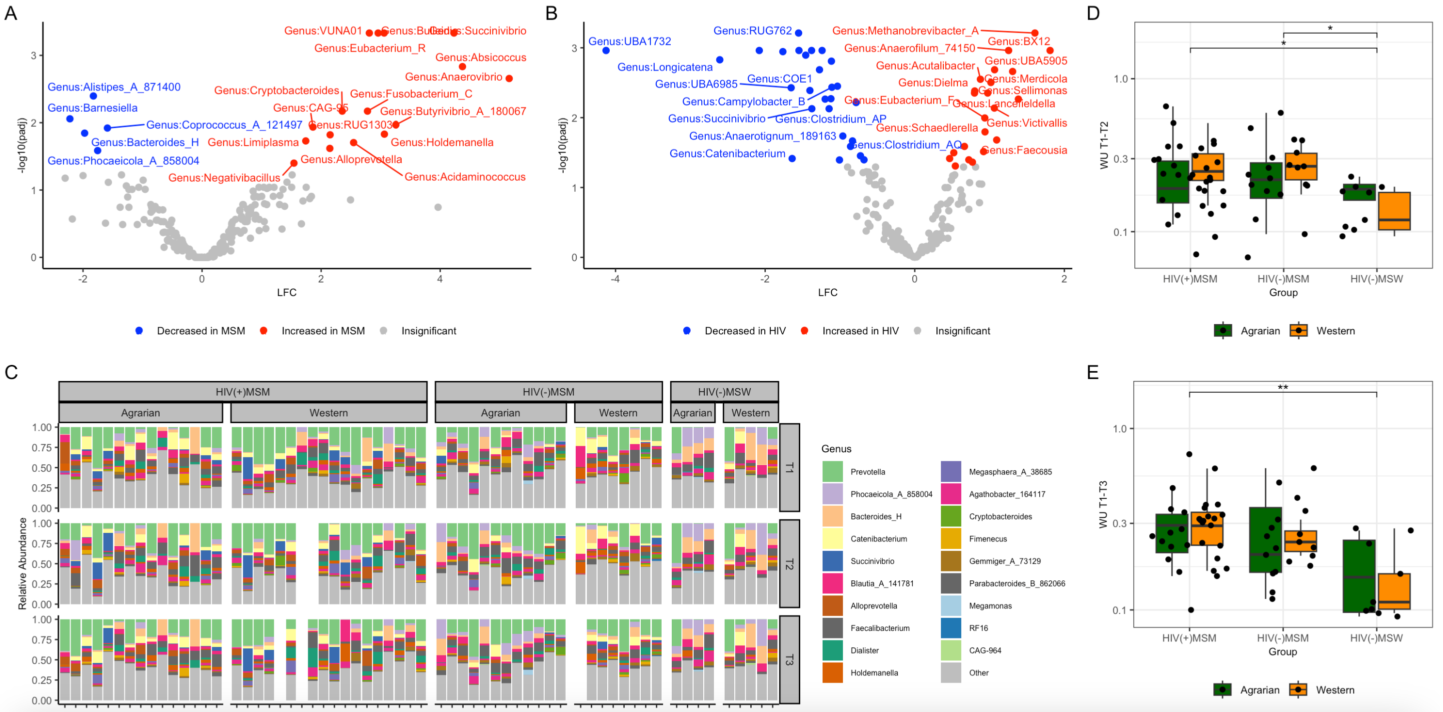

Figure S4: Fecal Microbiomes Differ by HIV and MSM Status and Exhibited No Agrarian Diet-induced Changes. Analysis of Compositions of Microbiomes with Bias Correction (ANCOM-BC) results comparing fecal microbiome genera at baseline between (A) HIV(+) and HIV(-)MSM, (B) HIV(-)MSM and HIV(-)MSW; (C) Bar plots of most abundant genera stratified by group, diet, and timepoint; Weighted Unfrac distances from baseline to (D) T2 and (E) T3; groupwise statistics determined by Kruskal-Wallace with Dunn's post hoc test *-p≤0.05, **-p≤0.01, ***-p≤0.001, ****-p≤0.0001. LFC=log-fold change, HIV=human immunodeficiency virus, MSM=men who have sex with men, MSW=men who have sex with women.

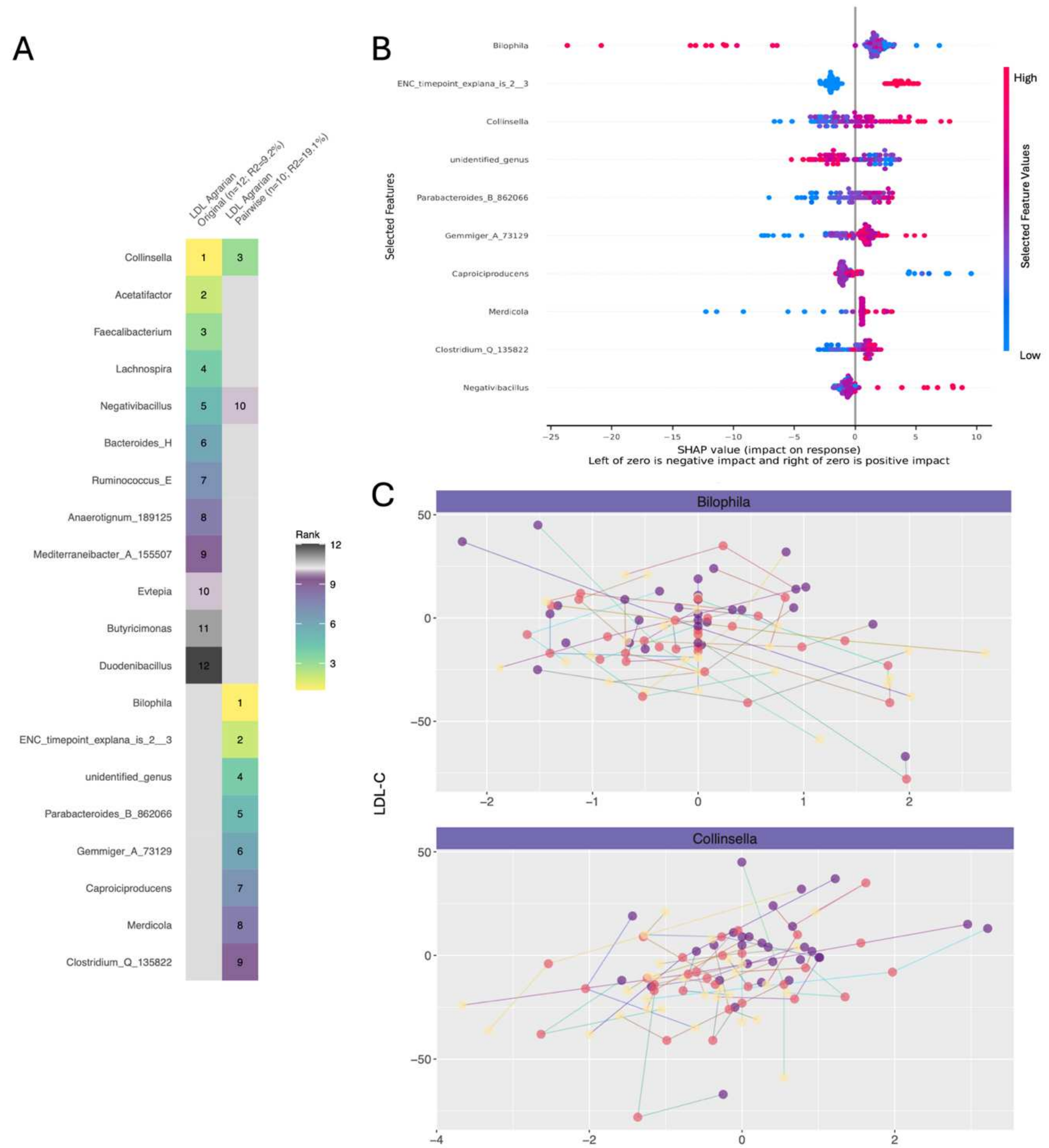

Figure S5. Features Related to LDL-C in Individuals on an Agrarian Diet Intervention, Selected Using Mixed-effects Random Forests. EXPLANA (EXPLoratory ANAlysis) software was used to find important features over three timepoints using original values and pairwise changes/deltas in features between timepoint per individual. A) Features selected using original data or pairwise comparisons are ranked, where one is most important. The top ten features per model are emphasized using a sequential, multi-hue, color palette from light to dark, and features after ten are grayscale from light to dark. *Collinsella* and *Negativibacillus* were selected by both models, while most genera were unique to original or pairwise. B) SHAP beeswarm plot of top ten features by magnitude of impact on LDL-C using model built from pairwise deltas. Each point represents one sample, and the horizontal position indicates impact on outcome as indicated on the x-axis. Points to the left indicate a negative impact, and points to the right indicate a positive impact. The colors represent feature values, where red is larger, and blue is smaller. For binary encoded categorical features (‘ENC’) such as timepoint, red is yes/1 and blue is no/0. C) Scatterplot showing pairwise differences in center log-ratio transformed *Bilophila* and *Collinsella* values (top two important genera selected using pairwise comparisons) with respect to LDL-C. Points are colored by timepoint 1_2 (yellow), 1_3 (red), and 2_3 (purple) and lines connect data per individual. Linear mixed-effects models using pairwise deltas confirm LDL-C has a negative relationship to *Bilophila* and a positive relationship to *Collinsella*. LDL-C=low density lipoprotein cholesterol.

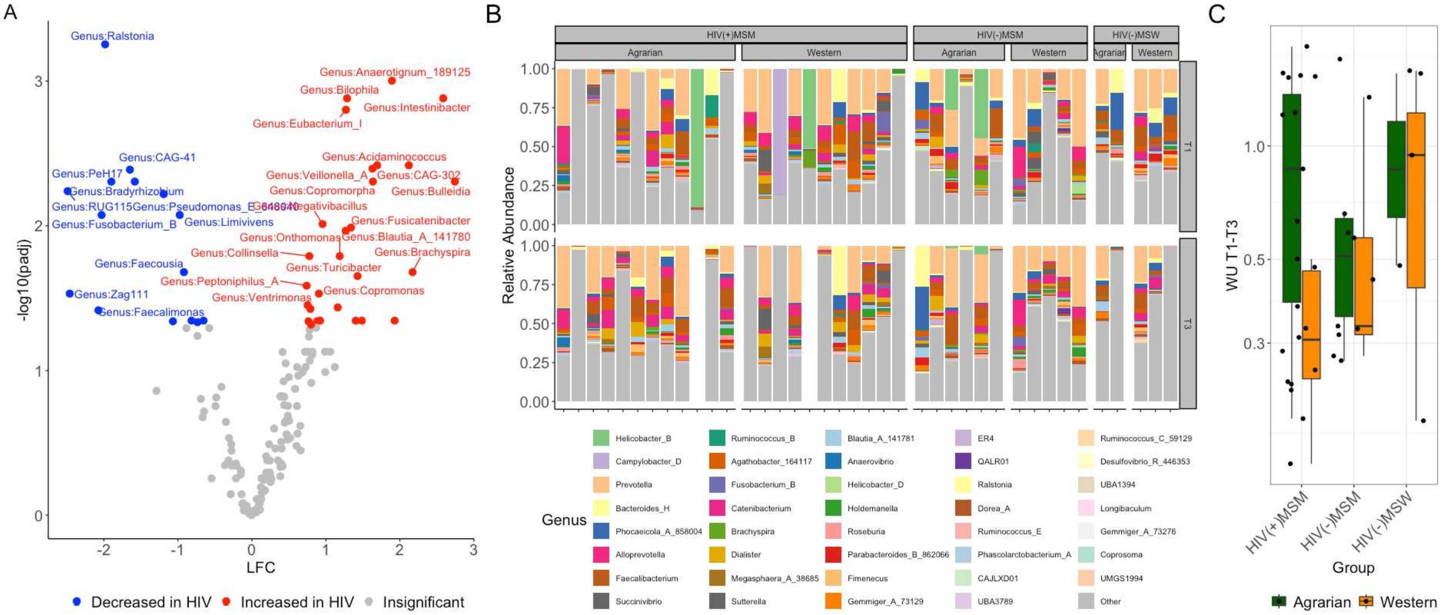

Figure S6: Biopsy Microbiomes Differ by HIV and Exhibited No Agrarian Diet-induced Changes. ANCOM-BC results comparing biopsy microbiome genera at baseline between (A) HIV(+) and HIV(-)MSM; (B) Bar plots of most abundant genera stratified by group, diet, and timepoint; (C) Weighted UniFrac distances from baseline to T3. LFC=log-fold change, HIV=human immunodeficiency virus, MSM=men who have sex with men, MSW=men who have sex with women.

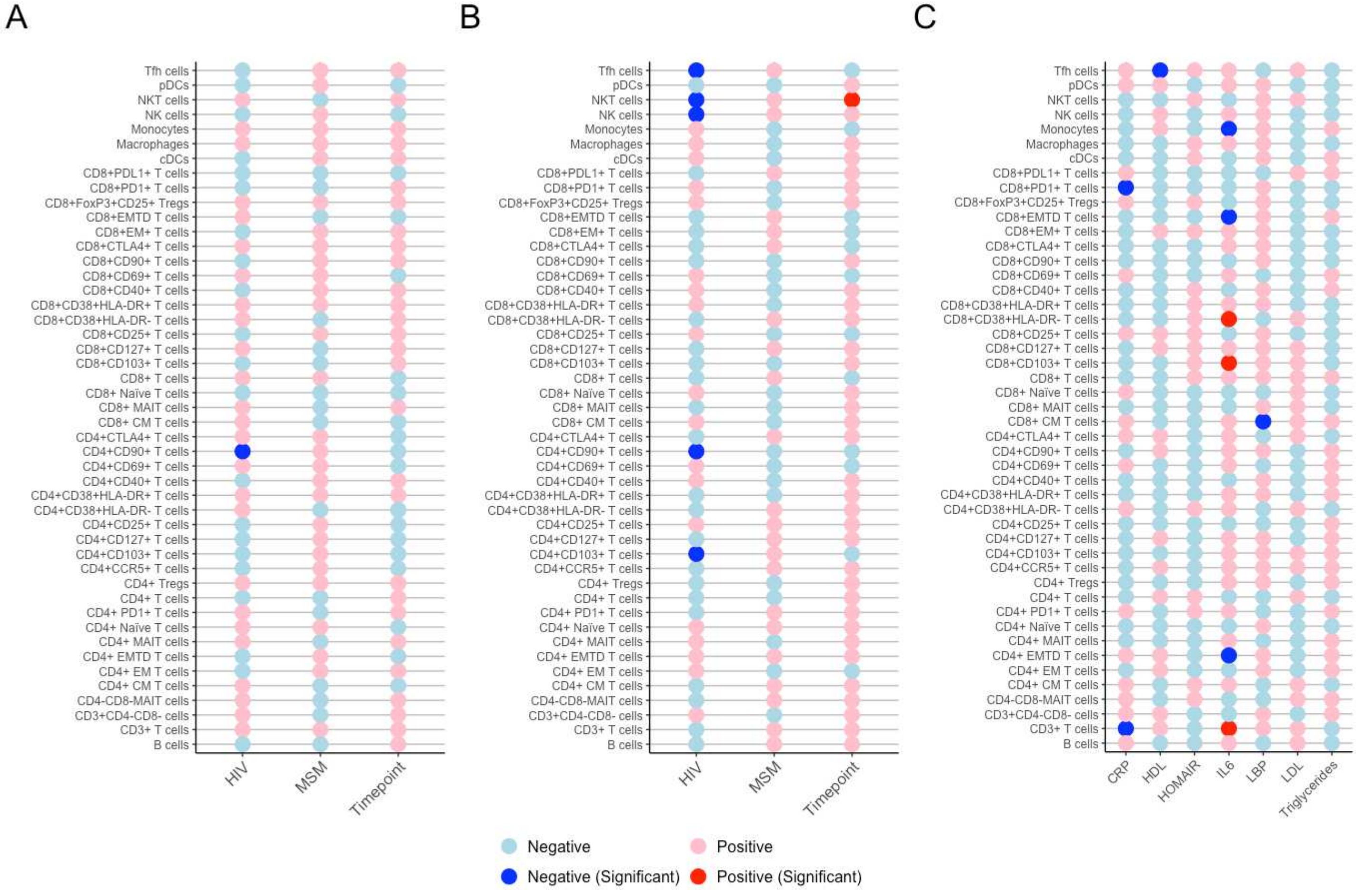

Figure S7: AD Associated with No Changes in Biopsy Immune Cell Populations. Coefficients of linear mixed-effects models (LMEMs) relating biopsy immune cells to MSM status, HIV status, and time in (A) those on the AD and (B) those on the WD. Red indicates a positive relationship with positive HIV status, MSM status, and increases while blue indicates the opposite. P-values determined by analysis of variance (ANOVA) of full model Cell Population ~ HIV+MSM+(1|StudyID) vs model removing predictor of interest. Significance determined by a p-value under 0.05. (C) coefficients of LMEMs comparing each cell population to inflammatory and metabolic markers. P-values determined by ANOVA of full model (Cell Population ~ CRP+HDL+HOMAIR+IL-6+LBP+LDL+Triglycerides+HIV+MSM+Diet(1|StudyID) vs model removing predictor of interest. Diet, MSM, and HIV coefficients not displayed. LDL-C=low-density lipoprotein cholesterol, HDL-C=high-density lipoprotein cholesterol, LBP=lipopolysaccharide binding protein, HOMA-IR=Homeostatic Model Assessment for Insulin Resistance, IL-6=Interleukin-6, CRP=C reactive protein.
